# Exploring pleiotropy using principal components

**DOI:** 10.1186/1471-2156-4-S1-S53

**Published:** 2003-12-31

**Authors:** Jeannette T Bensen, Leslie A Lange, Carl D Langefeld, Bao-Li Chang, Eugene R Bleecker, Deborah A Meyers, Jianfeng Xu

**Affiliations:** 1 Center for Human Genomics, Wake Forest University School of Medicine, Winston-Salem, North Carolina, USA; 2 Department of Public Health Sciences, Wake Forest University School of Medicine, Winston-Salem, North Carolina, USA

**Keywords:** multivariate analysis, principle components, quantitative traits, heritability estimates, linkage

## Abstract

A standard multivariate principal components (PCs) method was utilized to identify clusters of variables that may be controlled by a common gene or genes (pleiotropy). Heritability estimates were obtained and linkage analyses performed on six individual traits (total cholesterol (Chol), high and low density lipoproteins, triglycerides (TG), body mass index (BMI), and systolic blood pressure (SBP)) and on each PC to compare our ability to identify major gene effects. Using the simulated data from Genetic Analysis Workshop 13 (Cohort 1 and 2 data for year 11), the quantitative traits were first adjusted for age, sex, and smoking (cigarettes per day). Adjusted variables were standardized and PCs calculated followed by orthogonal transformation (varimax rotation). Rotated PCs were then subjected to heritability and quantitative multipoint linkage analysis. The first three PCs explained 73% of the total phenotypic variance. Heritability estimates were above 0.60 for all three PCs. We performed linkage analyses on the PCs as well as the individual traits. The majority of pleiotropic and trait-specific genes were not identified. Standard PCs analysis methods did not facilitate the identification of pleiotropic genes affecting the six traits examined in the simulated data set. In addition, genes contributing 20% of the variance in traits with over 0.60 heritability estimates could not be identified in this simulated data set using traditional quantitative trait linkage analyses. Lack of identification of pleiotropic and trait-specific genes in some cases may reflect their low contribution to the traits/PCs examined or more importantly, characteristics of the sample group analyzed, and not simply a failure of the PC approach itself.

## Background

Principal component analyses often provide valuable information that allows data reduction and reveals relationships between variables that were not previously suspected. As we begin to better understand the scope of gene effects, we find that single genes often contribute to multiple phenotypes (pleiotropy). Therefore, when mapping genes for complex disorders, it can be helpful to identify groups of variables or phenotypes (principal components) that may be controlled by a single gene. Arya et al. [1] demonstrated the practical application of principal component analysis by evaluating eight insulin resistance syndrome-related phenotypes in 27 nondiabetic Mexican-American extended families [1]. In their analyses, they identified three principal components factors and following multipoint variance components linkage analyses, their adiposity-insulin factor showed linkage at two different regions on chromosome 6q with LOD scores > 4.1. This observation was consistent with their previous finding of a major susceptibility locus for insulin resistance on chromosome 6q, which has been shown to have strong pleiotropic effects on other insulin resistance syndrome-related phenotypes such as body mass index (BMI) and leptin levels [1,2]. To examine this type of pleiotropic gene effect seen in the Arya study, we chose to evaluate the use of standard principal components (PC) methods to capture this effect in the Genetic Analysis Workshop (GAW13) simulated data set. Our first objective was to assess whether the traits grouped together in one of the PCs in our data analysis actually correspond to traits that share common gene effects in the underlying GAW13 simulated genetic model, our second objective was to identify the heritability of these PCs, and our third objective was to identify major pleiotropic genes through linkage analysis.

## Methods

All analyses were performed on the simulated data without missing observations. Replicate data set 57 was randomly selected and analysis was limited to the year 11 time point. Year 11 was selected because this was the first year in which Cohorts 1 and 2 both had data collected. Observations with triglyceride values greater than 400 (*n *= 27) were excluded in order to obtain valid low density lipoprotein (LDL) calculations. In addition, several (*n *= 8 > 4 SD) observations were excluded because they were judged to be highly influential in the PC analysis.

PC analysis was conducted on six quantitative traits (QTs): total cholesterol (Chol), triglycerides (TG), high density lipoprotein (HDL), LDL, systolic blood pressure (SBP), and body mass index (BMI). LDL was calculated using the Friedewald's equation [3]: (Total Chol - HDL) - TG/5, where TG = 400. BMI was calculated as (weight (lb) / (height (in))^2^] * 703. Three of the QTs (Chol, TG, and SBP) were log-transformed in order to better conform to a normal distribution. Each QT was then regressed on sex, age, and cigarettes per day using linear regression modeling, and residuals were obtained. The residuals for each QT were then standardized. PCs were calculated from the correlation matrix of the standardized residuals corresponding to the six QTs using standard methods, in which all individuals are assumed to be independent. PC analysis was performed using PROC FACTOR in the SAS statistical software package (version 8.2, Cary, NC), with PC extraction and varimax rotation (Table 1). Results from this analysis were used to create PCs consisting of linear combinations of individual QT residuals.

Heritability estimation and quantitative multipoint linkage analysis were performed on the PCs and on the residuals for individual QTs using variance-component methodology, as implemented in the Sequential Oligogenic Linkage Analysis Routines (SOLAR) [4]. Genotype data provided from all individuals were used to generate multipoint identity-by-descent (IBD) estimates throughout the genome. Phenotypic traits examined included the PCs and the raw QTs. No additional covariate adjustment was made at this stage. All analyses were performed a second time, with additional adjustment for cohort effect (using an indicator variable) when residuals were obtained. This was done in order to examine whether cohort had an effect after adjusting for age.

We did not consult the GAW13 simulated data set answers prior to either the interpretation of the PCs or performing linkage analysis. Verification of genes modeled in the simulated data set at baseline (not those influencing longitudinal data) were considered verified if linkage analysis identified a marker with a peak LOD score (LOD > 1.0) within 20 cM of the gender-averaged chromosomal location for a simulated trait gene. While there is little consensus regarding the most appropriate LOD score threshold for complex disease, similar to other studies of complex disease reporting LODs less than 2.0, we considered LOD scores greater than 1.0 as suggestive evidence of linkage [5,6].

## Results

At year 11 we had complete data on 989 individuals (316 families) from Cohort 1, mean age 59.9 years, and 1511 individuals (330 families) in Cohort 2, mean age 53.4. Variable means for the QTs and confounders were comparable between cohorts, except for SBP, TGs, and cigarettes per day, where mean SBP and TG were higher in Cohort 1 than 2 (SBP: 137 vs. 130 and TG: 146 vs. 136, respectively) and mean cigarettes per day were lower in Cohort 1 than 2 (4 vs. 6, respectively). After adjustment for age, sex, and cigarettes per day, cohort was a statistically significant predictor of only one of the QTs: SBP. The additional adjustment for cohort produced results (PCs and linkage) that were similar to those reported and did not change any of our conclusions.

The first three principal components identified in this analysis contributed to 73% of the overall phenotypic variance among the six QTs (Table 2). Heritability estimates (polygenic) for individual QTs and the three primary principal components were all statistically significant (*p *< 0.0001), ranging from 0.60 for LDL to 0.79 for BMI (Table 2). Standard errors for the heritabilities for all QTs and PCs were typically between 0.03 and 0.04

For PCs, linkage analysis only yielded LOD scores greater than 1.0 but less than 2.0 for PC3 (SBP + 2/3 BMI). Two of the three LODs in this range were false-positive results according to our criteria, while the third LOD identified a minor gene (b10) contributing 1% of trait variation for height (Table 3).

For individual traits, no LOD scores > 1.0 were observed for log Chol, HDL, LDL, or log SBP (Table 4). Log TG yielded two LOD scores between 1.0 and 2.0, both of which were false-positive findings, while BMI produced 31 LOD scores > 1.0, with 4 scores > 2.0. When considering the LODs between 1.0 and 2.0 for BMI, 26 of 27 (96%) were false-positive results, while 1 LOD score identified a gene for height, a component of the BMI quantitative trait. Of the 4 LOD scores greater than 2 for BMI, 2 were false positive, 1 was essentially unrelated to the BMI trait identifying genes for cholesterol and HDL, while only the highest LOD (5.4) identified a gene contributing 40% to trait variance for weight.

Table 5 indicates the linkage results within 20 cM of the two pleiotropic genes, b12 and b13, that contribute the largest proportion to the phenotypic variance of both HDL and TG. No elevated LOD score > 1.0 was identified for either PC1, PC2, or PC3.

## Discussion

Pleiotropic effects are a common phenomenon in reported studies of complex disease. Methods are needed to identify pleiotropic genes that may contribute differing amounts to the variances of multiple phenotypes. To this end, we chose to evaluate our ability to identify such genes by PC analysis, followed by heritability estimates and linkage analysis.

While our analysis was somewhat limited in terms of the number of variables available in the *complete *data set, PC analysis of the six variables identified three primary PCs explaining 79% of the phenotypic variance. Covariates (age, gender, and smoking) were adjusted *prior *to PC analysis, consistent with the strategy used by Moser et al., although concerns about the effect of these adjustments on PC and heritability estimates arose [7]. We therefore performed covariate adjustments *before *and *after *PC analysis [data not shown] and found no significant differences in PCs, loading, or heritability. Overall, the PC analysis, in particular PC2, reflected the pleiotropic genes (HDL and TG) modeled in the simulated data.

Heritability estimates were statistically significant for each of the three major PCs, as were those for the traits evaluated individually. Each PC heritability estimate was consistent in magnitude with the trait heritabilities comprising the PC. PC2, which reflected the simulated model best with respect to shared gene effects, had a heritability estimate slightly higher than the two individual variables (HDL and TG) in the PC and closer to that for BMI alone. This higher heritability estimate for PC2 may reflect the accuracy with which PC identifies/groups variables with common genetic influence or it may reflect the significant influence of BMI on this PC.

Several factors that may have contributed to limited power in both our individual trait and PC linkage analyses include sample size and composition (single replicate), pedigree structure, and the number and size of genetic effects. One of the challenges facing linkage mapping for complex disease traits is adequate sample size. Risch and Merikangas state that the power of linkage for complex disease is limited to the detection of only the strongest loci unless thousands of small families are utilized [8]. In this report a total of 646 families were analyzed and thus may not have provided ample power for the detection of genes contributing modestly to trait variance. The analysis of a single replicate in the GAW13 simulated data set may also have hindered our ability to detect meaningful linkage.

Studies have shown the PC approach may improve the power to identify genes with pleiotropic effects involved in complex disease [1,9,10]. While PC heritability estimates were encouraging, we were unable to identify pleiotropic genes. One very plausible explanation may be that rather than a single gene with a major effect, the high heritability reflected many genes with small effects. While it has been shown that the PC approach has greater power to detect major pleiotropic genes [10], the power to detect genes with small effects is likely to be limited. In addition, our investigation was highly dependent on the extent of pleiotropy modeled in the simulated data set as well as our selection of variables for analysis. HDL, TG, and glucose were modeled as pleiotropic traits; however our investigation only considered HDL and TG (major components of our PC2). Ideally, PC2 would have identified at least the b12 gene contributing 20% and 10% to the variance of HDL and TG, respectively. Several investigators have demonstrated increased power and precision in identifying genetic effects when using multivariate approaches for correlated traits [11,12]. However, in a recent commentary, Meigs points out that the results of such analyses can be influenced by both the number and nature of variables included in the model [13]. The lack of our ability to identify the b12 gene in this simulated data set may have been due to the omission of glucose from our model or may reflect the difficulty our method has in identifying complex trait genes. Finally, while we utilized the standard PC method and adjusted for covariates prior to linkage analysis to maximize power, we may have missed potentially important genetic effects by focusing first on the PCs that explained the majority of phenotypic variation.

In summary, PC analysis has been demonstrated in reported studies of complex disease to localize regions of the human genome likely to contain pleiotropic genes [1], but may be influenced by factors such as the number and effect size of pleiotropic genes involved as well as complex trait variables available for inclusion in the PC analysis. Further studies are needed to assess the utility of the PC approach in complex disease.

## Figures and Tables

**Table 1:** Principal component trait loading values (rotated values).

**Trait**	**PC1**	**PC2**	**PC3**
**Log Chol**	0.96	0.03	0.08
**HDL**	-0.17	-0.73	0.21
**LDL**	0.99	0.01	-0.03
**Log TG**	-0.11	0.79	0.16
**BMI**	-0.04	0.44	0.54
**Log SBP**	0.07	-0.15	0.83

Major determinants of the PC were considered traits with loading values = 0.30.

**Table 2:** Variance and heritability estimates for individual traits and principal components.

	**Trait**	**Mean (SD)**	**H2r^A ^(SE)**
**1**	Log Chol	5.32 (0.17)	0.63 (0.04)
**2**	HDL	50.22 (11.64)	0.71 (0.03)
**3**	LDL	128.89 (37.90)	0.60 (0.04)
**4**	Log TG	4.83 (0.48)	0.62 (0.04)
**5**	BMI	26.74 (4.79)	0.79 (0.03)
**6**	Log SBP	4.88 (0.12)	0.75 (0.03)
**PC 1**	1/2 (Log Chol + LDL)		0.62 (0.04)
**PC 2**	1/2 BMI + (Log TG - HDL)		0.80 (0.03)
**PC 3**	Log SBP + 2/3 BMI		0.74 (0.03)

^A^H2r represents the polygenic contribution and H2q1 the contribution of major gene (H2r + H2q1 = overall heritability).

**Table 3:** Genome-wide linkage results for principal components.

Component	Peak	Maximum LOD	Chromosome	Position (cM)	Marker
		
PC 1	- - -	No LOD > 1.0			
PC 2	- - -	No LOD > 1.0			
PC 3	Peak 1	1.18	3	132	False +
	Peak 2	1.07	7	137	b10 @ 124 (height)
	Peak 3	1.16	15	20	False +

**Table 4:** Genome-wide linkage results for individual traits.

Trait	Peak	Maximum LOD	Chromosome	Position (cM)	Marker
		
Log Chol	- - -	No LOD > 1.0			
HDL	- - -	No LOD > 1.0			
LDL	- - -	No LOD > 1.0			
Log TG	Peak 1	1.55	12	166	False +
	Peak 2	1.02	19	68	False +
Log SBP		No LOD > 1.0			
BMI	Peak 8	3.11	4	35	False +
	Peak 19	2.74	11	50	b30 @66 (Chol) b21 @45 (HDL)
	Peak 23	5.40	13	55	b11 @70 (Weight)
	Peak 26	2.21	15	15	False +

TG = triglycerides, Chol = Cholesterol, SBP = systemic blood pressure, BMI = body mass index

**Table 5:** Unblinded major pleiotropic genes influencing TG and HDL-linkage results.

	**PC 1**			**PC 2**			**PC 3**		
	1/2 (Log Chol + LDL)			PC 2 1/2 BMI + (Log TG - HDL)			PC 3 Log SBP + 2/3 BMI		
	
**Gene**	**Max LOD**^**A**^	**H2r**^**B**^	**H2q1^B^**	**Max LOD**^**A**^	**H2r^B^**	**H2q1^B^**	**Max LOD**^**A**^	**H2r^B^**	**H2q1^B^**
**G(b12)**^**C**^	0.00	0.62	0.00	0.00	0.80	0.00	0.42	0.71	0.05
**G(b13)**^**D**^	0.00	0.62	0.00	0.00	0.80	0.00	0.24	0.72	0.03

^A^MaxLOD, the maximum LOD score within approximately 20 cM of the gene.

^B^H2r represents the polygenic contribution and H2q1 the contribution of major gene (H2r + H2q1 = overall heritability).

^C^G(b12) is located on chromosome 9 at 11 cM (MaxLOD range: 0 cM-35 cM).

^D^G(b13) is located on chromosome 9 at 83 cM (MaxLOD range: 65 cM-105 cM).
